# Dual-Hand Motion Capture by Using Biological Inspiration for Bionic Bimanual Robot Teleoperation

**DOI:** 10.34133/cbsystems.0052

**Published:** 2023-09-13

**Authors:** Qing Gao, Zhiwen Deng, Zhaojie Ju, Tianwei Zhang

**Affiliations:** ^1^ School of Electronics and Communication Engineering, Shenzhen Campus of Sun Yat-sen University, Shenzhen, China.; ^2^ School of Computing, University of Portsmouth, Portsmouth, UK.; ^3^ Shenzhen Institute of Artificial Intelligence and Robotics for Society, Chinese University of Hong Kong, Shenzhen, China

## Abstract

Bionic bimanual robot teleoperation can transfer the grasping and manipulation skills of human dual hands to the bionic bimanual robots to realize natural and flexible manipulation. The motion capture of dual hands plays an important role in the teleoperation. The motion information of dual hands can be captured through the hand detection, localization, and pose estimation and mapped to the bionic bimanual robots to realize the teleoperation. However, although the motion capture technology has achieved great achievements in recent years, visual dual-hand motion capture is still a great challenge. So, this work proposed a dual-hand detection method and a 3-dimensional (3D) hand pose estimation method based on body and hand biological inspiration to achieve convenient and accurate monocular dual-hand motion capture and bionic bimanual robot teleoperation. First, a dual-hand detection method based on body structure constraints is proposed, which uses a parallel structure to combine hand and body relationship features. Second, a 3D hand pose estimation method with bone-constraint loss from single RGB images is proposed. Then, a bionic bimanual robot teleoperation method is designed by using the proposed hand detection and pose estimation methods. Experiment results on public hand datasets show that the performances of the proposed hand detection and 3D hand pose estimation outperform state-of-the-art methods. Experiment results on a bionic bimanual robot teleoperation platform shows the effectiveness of the proposed teleoperation method.

## Introduction

With the rapid development of robotics in recent years, robots can replace or assist humans to complete some specific tasks in the fields of industry, medical care, aerospace, and educational services [1]. Since the structures of the bionic bimanual robots [2–4] are similar to that of the human dual arms and dual hands, they can replace humans to complete more complex tasks. The movement of the bionic bimanual robot requires a high degree of coordination and complex manipulation. Bionic bimanual robot teleoperation [5] can transfer the operation skills of the human dual hands to the robots to increase the success accuracy and reliability of the manipulation, which is the first choice for smart programming solution when dealing with fast decisions and corner cases.

**Fig. 1.**
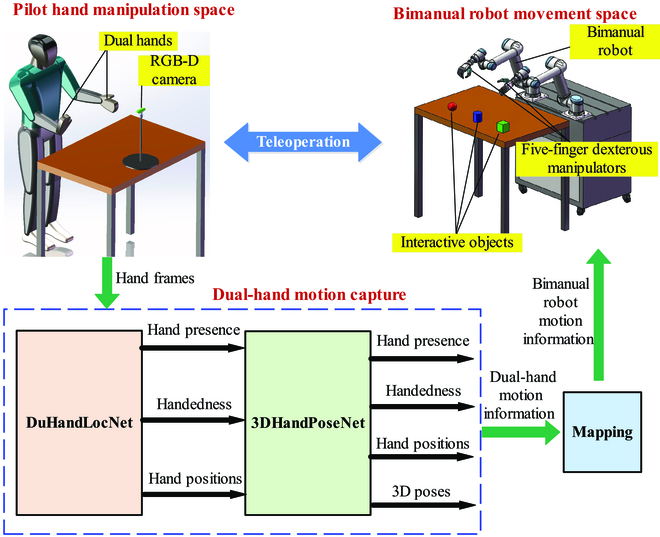
The pipeline of the bionic bimanual robot teleoperation.

Currently, teleoperation methods for degree-of-actuation robots like bionic bimanual robots require precise motion capture of human dual hands. Then, the captured motion information is mapped to the motion of robots to realize the teleoperation. The hand motion capture methods mainly include the methods based on data gloves [6], surface electromyography wristbands [7], and optical markers [8]. These methods are very expensive and require long and difficult calibration work. At present, there are already several commercialized software mark-free apps for motion capture, such as Kinect developed by Microsoft Co. several years ago that was a powerful and useful mark-free tool, and recently, numerous apps of motion capture for smartphones have been developed [9]. However, these apps have some disadvantages. For example, Kinect only has motion capture of the human body and does not include fine hand motion capture. Most apps for smartphones can only estimate 2-dimensional (2D) hand pose. These methods are not suitable for teleoperation of bionic bimanual robots. Based on these issues, this work provides a convenient, marker-free, and low-cost teleoperation strategy for bionic bimanual robots. The pipeline of the teleoperation method is shown in Fig. 1. The positions and poses of the pilot’s dual hands are captured by visual sensors. Then, the dual-hand motion information is mapped to the movement of the bionic bimanual robot. In this system, a visual dual-hand motion capture method is proposed, which includes a DuHandLocaNet and a 3DHandPoseNet. The DuHandLocaNet is used to detect and localize the dual hands to obtain the hand presence, handedness, and hand positions, which can be mapped to the end positions of the bionic bimanual robot. The 3DHandPoseNet is used to estimate the 3-dimensional (3D) poses of the dual hands from the located hand RGB images, and these 3D hand poses can be mapped to the gestures of the 5-finger dexterous manipulators.

The dual-hand motion capture plays an important role in the bionic bimanual robot teleoperation. However, it is still a great challenge in the computer vision. For one thing, the detection and distinction of dual hands are affected by the similarity of the left and right hands, multiple gestures, and small sizes. So, it is difficult to obtain high accuracies. For another, the 3D hand pose estimation from single RGB images is also difficult due to the self-similarity, self-occlusion, and lack of depth information. To deal with these issues, this work proposes a biologically-inspiration-based dual-hand motion capture method, which includes a dual-hand detection network (DuHandLocNet) and a 3D hand pose estimation network (3DHandPoseNet). The DuHandLocNet uses a parallel network structure to combine the features of the hands with the relationship between the dual hands and body. By introducing the biological constraints of the relationship between the dual hands and body into the loss function to increase the hand detection accuracy and distinguish the left and right hands. The 3DHandPoseNet adopts a cascaded structure to extract the joint point features of each hand part and introduces hand bone constraints into the loss function to improve the 3D hand pose estimation.

The contributions and innovations of this work are shown as follows.

1. A DuHandLocNet is proposed to deal with dual-hand detection and localization, which introduces the biological constraint of the structure relationship between the dual hands and body by using a parallel network.
2. A 3DHandPoseNet is proposed to deal with 3D hand pose estimation from single RGB images, which is based on the hand biological inspiration by using a cascaded structure and a bone-constraint loss.
3. A visual dual-hand-based teleoperation system for bionic bimanual robots is designed by using the proposed DuHandLocNet and 3DHandPoseNet. It can realize free and convenient teleoperation.

The remainder of this paper is organized as follows. Related work introduces related works. Dual-hand detection network and 3D Hand pose network introduce the proposed dual-hand detection method (DuHandLocNet) and 3D hand pose estimation method (3DHandPoseNet), respectively. Bionic bimanual robot teleoperation system introduces the bionic bimanual robot teleoperation system. Experiment results and validation are shown in Results and Discussion. The conclusion and future work are shown in Conclusion.

## Materials and Methods

### Related work

In the following, the works of visual hand-based teleoperation, dual-hand detection, and 3D hand pose estimation are discussed, which are closely related to our work.

### Visual hand-based teleoperation

Compared with the hand teleoperation methods based on data gloves [6], surface electromyography wristbands [7], and optical markers [8], the visual hand teleoperation is still immature. However, due to its huge potential, there are still some works that have made some progress. Li et al. [10] proposed an end-to-end neural network (TeachNet), which used a consistent error formula to control a Shadow hand, which is a 5-finger manipulator. Then, Li et al. [11] also proposed a multimodal mobile robot arm teleoperation system, which consists of a novel vision-based gesture regression network (Transteleop) and an inertial measurement unit-based arm tracking method. Handa et al. [12] designed a low-cost and vision-based teleoperating system (DexPilot), which can achieve full control of 23 degree of actuation’s robotic system by observing the bare hands. Gomez-Donoso et al. [13] designed a HandLocNet for hand detection and localization on RGB images and HandPoseNet for 3D hand pose estimation. It also conducted teleoperation experiments for AR10 and Shandow hands in a virtual environment. Sivakumar et al. [14] designed a Robotic Telekinesis for robotic hand teleoperation by watching humans on Youtube. These teleoperation methods are all used for single-arm/hand robots and not suitable for the bionic bimanual robots.

### Visual dual-hand detection and distinction

The visual dual-hand detection and distinction is a great challenge in computer vision due to the similarity of the left and right hands, the variety of gestures, and the small sizes of hands. Some traditional methods use hand color, optical flow, and shape features [15]; they rely heavily on limited conditions and lack of generalization in practical applications [16]. With the development of deep learning in object detection, some deep learning networks like Faster R-CNN [17], SSD [18], and YOLO [19] have been introduced to deal with hand detection and dual-hand distinction. For example, Hoang Ngan Le et al. [20] proposed Multiple Scale Region-based Fully Convolutional Networks to realize robust hand detection in vehicles. However, this method is only suitable for a few hand gestures such as the driving gestures. Gao et al. [21] improved the SSD to achieve robust real-time hand detection and localization. However, this method did not discuss the distinction of left and right hands. Gao et al. [22] also proposed a dual-hand detection method by using parallel network. This method requires postprocessing and cannot be used in a real-time system.

### 3D hand pose estimation from single RGB images

The 3D hand pose estimation from single RGB images is also a great challenge in computer vision due to the hand self-similarity, self-occlusion, and lack of depth information. To deal with this issue, Ge et al. [23] proposed a 3D hand pose estimation method by using 3 CNN subnetworks, which are used for hand segmentation, 2D hand pose detection, and 2D to 3D hand pose derivation, respectively. Ge et al. [24] also proposed an end-to-end trainable hand pose and mesh generation approach based on Graph CNN [25]. Lin et al. [26] proposed a MEsh TRansfOrmer method to reconstruct 3D hand pose and mesh vertices from a single image by using the transformer [27]. These methods are all directly transferred from the 3D body pose estimation methods. Although they have achieved high accuracy, they do not consider the structural features of the hand.

## Dual-hand detection network

**Fig. 2.**
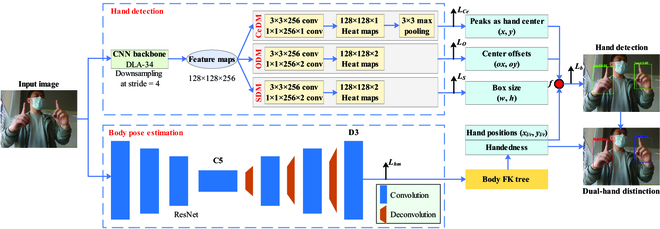
The pipeline of the DuHandLocNet.

The DuHandLocNet is designed for dual-hand detection and distinction on RGB images. The pipeline of the DuHandLocNet is shown in Fig. 2. It uses a parallel network, where one subnetwork is designed for hand detection and the other subnetwork is designed for body pose estimation, which can output the estimated dual-hand positions and handedness by using a body forward kinematic (FK) tree [22]. The estimated dual-hand positions can help to increase the hand detection accuracy by introducing it into the loss function. The handedness can help to distinguish left and right hands from the hand detection results.

### Hand detection subnetwork

The hand detection network adopts the anchor-free structure [28,29]. Compared with the anchor-based structure [17–19], there is no need to preset anchors. It only needs to regress the center point, width, and height of objects with different scales, which greatly reduces the consumption. In addition, the structure draws on the keypoint-based structure of CenterNet [29], which can improve the detection accuracies of hands with small sizes. In the network, the deep layer aggregation (DLA-34) [30] is adopted as the backbone. DLA-34 is one of the most advanced methods for image semantic segmentation currently. It utilizes separable convolution blocks to accelerate computation speed while preserving image semantic information. To apply it to hand detection, we add additional skip connections from the bottom layers to the original DLA-34 structure. Additionally, we upgrade each convolutional layer during the upsampling stage. Then, 3 models are followed as the heads, which are center detection module (CeDM), offset detection module (ODM), and size detection module (SDM). For an input image *I*, the CeDM is used to predict the hand center coordinates (*x*, *y*). The SDM is used to predict the hand size (*w*, *h*), and the ODM is used to predict the hand localization offset (*ox*, *oy*). As a result, the bounding box of the detected hand Bh can be formulated as $\left(\left[x-\frac{w}{2}+\mathrm{ox},x-\frac{h}{2}+\mathrm{oy}\right],\left[x+\frac{w}{2}+\mathrm{ox},x+\frac{h}{2}+\mathrm{oy}\right]\right)$.

For training, the CeDM uses the focal loss as its loss function *L_Ce_*; the equation is shown as

$$L_{\mathrm{Ce}}=\frac{1}{N}\;\sum_{i,j}\left\{\begin{array}{cc} {\left(1\;-Y_{i,j}\right)}^{\alpha }\text{log}\;\left(Y_{i,j}\right) & \text{if}\;{\hat{Y}}_{i,j}\;=\;1\\ {\left(1\;-\;{\hat{Y}}_{i,j}\right)}^{\beta }\;{\left(Y_{i,j}\right)}^{\alpha }\text{log}\;\left(1-Y_{i,j}\right) & \text{otherwise} \end{array}\right.$$ (1)

where *Y*_*i*, *j*_ is the score at point (*i*, *j*) in the heat map of the predicted hand center point, and ${\hat{Y}}_{i,j}$ is the corresponding ground truth. *N* is the hand number in the image *I*, and *α* and *β* are focal loss hyperparameters.

The SDM uses the *L1* regression loss as its loss function *L_S_*; the equation is shown as

$$L_{s}\;=\;\frac{1}{N}\;\sum_{i,j}\left(\left|\omega_{i,j}-{\hat{\omega }}_{i,j}\right|\;+\;\left|h_{i,j}-{\hat{h}}_{i,j}\right|\right)$$ (2)

where *ω*_*i*, *j*_ and *h*_*i*, *j*_ are the width and length of the predicted hand bounding box, and ${\hat{\omega }}_{i,j}$ and ${\hat{h}}_{i,j}$ are the corresponding ground truths.

The ODM also uses the *L1* regression loss as its loss function *L_O_*, and its equation is shown as

$$L_{O}\;=\;\frac{1}{N}\;\sum_{i,j}\left|O_{i,j}\;-\;\left(\frac{\hat{c}}{s}\;-\;c\right)\right|$$ (3)

where *c* is the predicted hand center point and $\hat{c}$ is the corresponding ground truth. *S* = 4 is the number of the downsampling. *O*_*i*, *j*_ is the predicted hand localization offset.

### Body pose estimation subnetwork

The structure of the proposed body pose estimation network draws on the structure in [31], which introduces 3 deconvolution layers behind the C5 layer of the ResNet [32]. These deconvolution layers all use ReLU activation and batch normalization. Each layer has a 4 × 4 convolution kernel and 256 filters. Then, a 1 × 1 convolution layer is introduced at the end of the network to generate the predicted heatmaps *h*_1_, *h*_2_, …, *h_k_* for the *k* body joint points. The heat-map loss *L_hm_* uses the mean squared error, and its equation is shown as

$$L_{\mathrm{hm}}\;=\;\sum_{i,j}^{k}\;{\left\|h_{j}\;-\;{\hat{h}}_{j}\right\|}_{2}^{2}$$ (4)

where *j* ∈ [1, *k*] is means the *j*th joint point. *h_j_* and ${\hat{h}}_{j}$ are the heatmaps of the predicted *j*th joint point and the corresponding ground truth, respectively.

**Fig. 3.**
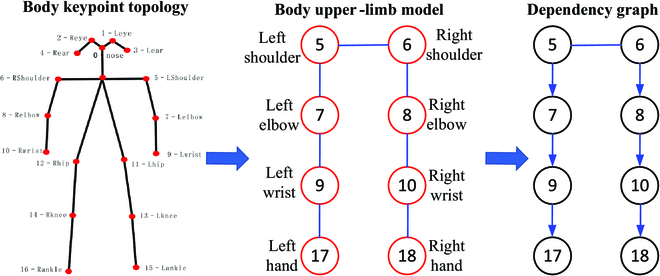
Dependency graph. The left image is body keypoint topology, which includes 10 keypoints. The middle image is body upper-limb model, where the keypoints of left hand (keypoint 17) and right hand (keypoint 18) are 2 added keypoints. The right image is the dependency graph obtained from body upper-limb model.

As shown in Fig. 2, since the body keypoint topology has no dual-hand joint points, the body FK tree is used to estimate the coordinates of the left and right hands according to the body skeletal structure. As shown in Fig. 3, first, the body upper-limb model is made up of the upper-limb joint points from the body keypoint topology and defined left and right joint points (17 and 18). Then, the dependency graph including parent and child nodes are built according to the upper-limb model. So, the dual-hand joint points P17 and P18 can be obtained from the dependency relationship between the parent and child nodes.

$$\left\{\begin{array}{c} P_{17}=s\cdot h_{7,9}+P_{9}\\ P_{18}=s\cdot h_{8,10}+P_{10} \end{array}\right.$$ (5)

### Outputs

From the hand detection subnetwork, the hand center point coordinate (*P_h_*) can be obtained. From the body pose estimation subnetwork, the dual-hand joint coordinates and their handedness (*P_l_* = *P*_17_, *P_r_* = *P*_18_) can be obtained. Then, a biological constraint loss *L_b_* is introduced, which is shown as follows.

$$L_{b}=\min\left(\left|P_{h}-P_{l}\right|,\left|P_{h}-P_{r}\right|\right)$$ (6)

The *L_b_* represents the biological structure relationship between the dual hands and the body.

Therefore, the total loss function *L* is defined as

$$L=L_{\mathrm{Ce}}+L_{S}+L_{O}+L_{b}$$ (7)

Then, the distinction between left and right hands is determined by the position relationship between the coordinates of the detected hand center point and the estimated dual hands. Its equation is shown as follows.

$$\text{Handedness}=\left\{\begin{array}{ll} \text{Lefthand} & \text{if}\left(\left|P_{h}-P_{l}\right|\leq \left|P_{h}-P_{r}\right|\right)\\ \text{Righthand} & \text{if}\left(\left|P_{h}-P_{l}\right|>\left|P_{h}-P_{r}\right|\right) \end{array}\right.$$ (8)

## 3D Hand pose network

**Fig. 4.**
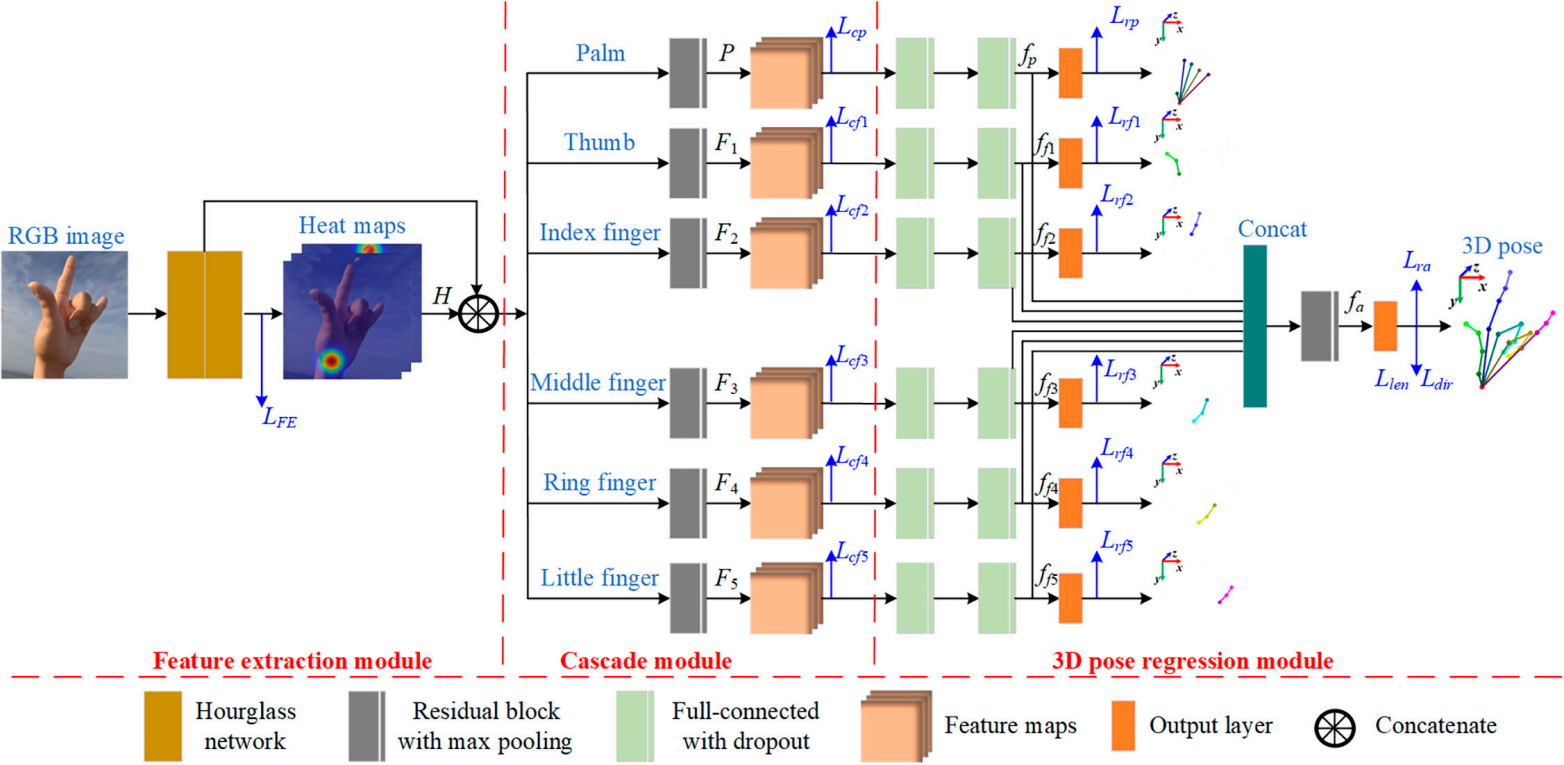
The structure of the 3DHandPoseNet.

After the dual-hand detection, the located hand RGB image is input to the proposed 3DHandPoseNet to obtain the 3D hand pose. To improve the 3D hand pose estimation performance, the network is introduced a cascaded structure and a bone-constraint loss. The structure of the network is shown in Fig. 4, which includes a feature extraction module (FEM), a cascade module (CaM), and a 3D pose regression module (PRM). The FEM is used to extract the 2D hand joint point features, the CaM is used to fine-tune the 2D hand joint point features, and the PRM is used to regress the 3D hand pose. The detailed introduction is shown as follows.

### Feature extraction module

The FEM uses 2 stacked hourglass modules [33] as its backbone to extract the 2D hand joint (2D hand pose) features. The 2D heat maps of 21 hand joints are used as the output of the FEM, which not only improves the generalization ability but also reduces the learning capacity. The heat-map loss *L_FE_* is utilized for training, and its loss function is formulated as

$$L_{\mathrm{FE}}=\sum_{n=1}^{A}\sum_{u,v}{\left\|\hat{H_{n}^{a}}\left(u,v\right)-H_{n}^{a}\left(u,v\right)\right\|}^{2}$$ (9)

where *A* is the number of hand joint points. $H_{n}^{a}$ and $\hat{H_{n}^{a}}$ are the estimated and ground-truth 2D heat maps of the *n*th hand joint point, respectively. The 2D heat map resolution is 64 × 64 pixels. (*u*, *v*) is the coordinate in the image coordinate system. After the feature extraction, the extracted feature maps are concatenated with the heat maps and the concatenated feature maps are output to the CaM.

### Cascade module

It can be found by analyzing the structure of the hand that the motions of joint points in different fingers and palm are independent, while the motions of joint points in the same finger or palm are dependent. Therefore, as shown in Fig. 4, the cascade structure is proposed to divide the feature extraction into multiple subnetworks, which are palm, thumb, index finger, middle finger, ring finger, and little finger subnetworks. By using this cascade structure, more appropriate weight parameters for hand joint feature maps can be obtained. In each subnetwork, a residual block with a max-pooling layer is used to fine the feature maps. The cascade loss function *L_cs_* (*s* = *p*, *f*1, *f*2, …, *f*5) is formulated as

$$L_{\mathrm{cs}}\;=\;\sum_{n=1}^{S}\sum_{u,v}{\left\|\hat{H_{n}^{s}}\;\left(u,v\right)-H_{n}^{s}\left(u,v\right)\right\|}^{2}$$ (10)

where *S* is the number of joint points of palm (*P*) and 5 fingers (*F1* to *F5*). As shown in Fig. 4, *P* contains 6 joint points, and *F1* to *F5* contain 3 joint points, respectively. $H_{n}^{s}$ and $\hat{H_{n}^{s}}$ are estimated and ground-truth heat maps, respectively. (*u*, *v*) is the coordinate in the image coordinate system.

Therefore, the total loss function *L_Ca_* in the CaM is formulated as

$$L_{\mathrm{Ca}}=L_{\mathrm{cp}}+L_{\mathrm{cf}1}+\cdots +L_{\mathrm{cf}5}$$ (11)

### 3D Pose regression module

The refined feature maps from the CaM are input to the PRM to regress the 3D hand joint points (3D hand pose). The PRM also uses the cascade structure to regress the fingers and palm joint points in 6 subnetworks. As shown in Fig. 4, each subnetwork uses 2 full-connected layers with dropout. In addition, the feature maps from all subnetworks are concated and input to another full-connected layer with dropout to obtain the 3D pose of the entire hand. The regression loss function *L_rs_* (*s* = *a*, *p*, *f*1, …, *f*5, ) is formulated as

$$L_{\mathrm{rs}}\;=\;\sum_{n=1}^{S}{\left\|\hat{J_{n}^{s}}-J_{n}^{s}\right\|}_{2}^{2}$$ (12)

where *S* is the number of joint points of the entire hand (*A*), palm (*P*), and 5 fingers (*F1* to *F5*). As shown in Fig. 4, *A* contains 21 joint points, *P* contains 6 joint points, and* F1* to *F5* contain 3 joint points, respectively. $J_{n}^{s}$ and $\hat{J_{n}^{s}}$ are estimated and ground-truth 3D hand joint coordinates. Therefore, the sum of these regression loss function *L_PR_* is formulated as

To improve the 3D hand pose estimation performance, bone-constraint losses are also introduced to the loss functions. By analyzing the hand bone structure, it can be found that even though the errors of the hand joint points are small, there is a case where the errors of the bone lengths and the bone orientations are large. Therefore, 2 bone-constraint losses are proposed in the training stage, which are bone length and the bone orientation losses. The bone length loss function *L_len_* imposes translation constraints on the joints to provide a more rigid and natural hand skeletal structure, which is formulated as

$$L_{\mathrm{len}}=\sum_{i,j}\left|{\left\|\hat{b_{i,j}}\right\|}_{2}-\;{\left\|b_{i,j}\right\|}_{2}\right|$$ (14)

where *b*_*i*, *j*_ = *J_i_* − *J_j_* is the estimated bone vector between joint *i* and *j*, and $\hat{b_{i,j}}$ is the corresponding ground-truth bone vector. The bone orientation loss imposes rotational constraints on the joints so that the estimated hand pose looks undistorted. Its loss function *L_dir_* is formulated as

$$L_{\mathrm{dir}}\;\sum_{i,j}\left\|\frac{\hat{b_{i,j}}}{{\left\|\hat{b_{i,j}}\right\|}_{2}}-\frac{b_{i,j}}{{\left\|b_{i,j}\right\|}_{2}}\right\|$$ (15)

As a result, the total loss function *L_pose_* of the 3DHandPoseNet in the training stage is formulated as

$$L_{\text{pose}}=\lambda_{\mathrm{hm}}\left(L_{\mathrm{FE}}+L_{\mathrm{Ca}}\right)+\lambda_{\mathrm{pr}}L_{\mathrm{PR}}+\lambda_{\mathrm{len}}L_{\mathrm{len}}+\lambda_{\mathrm{dir}}L_{\mathrm{dir}}$$ (16)

where *λ_hm_*, *λ_pr_*, *λ_len_*, and *λ_dir_* are factors for the trade-off between the above losses.

## Bionic bimanual robot teleoperation system

The structure of the bionic bimanual robot teleoperation system proposed in this work is shown in Fig. 1. It includes a pilot hand manipulation space, a dual-hand motion capture module, a mapping module, and a bionic bimanual robot movement space.

### Pilot hand manipulation space

In the pilot hand manipulation space, a pilot can operate with dual hands by observing the motion state of the bionic bimanual robot. A RGB-D camera is used to capture the RGB and depth frames of the pilot’s dual hands.

### Dual-hand motion capture module

**Fig. 5.**
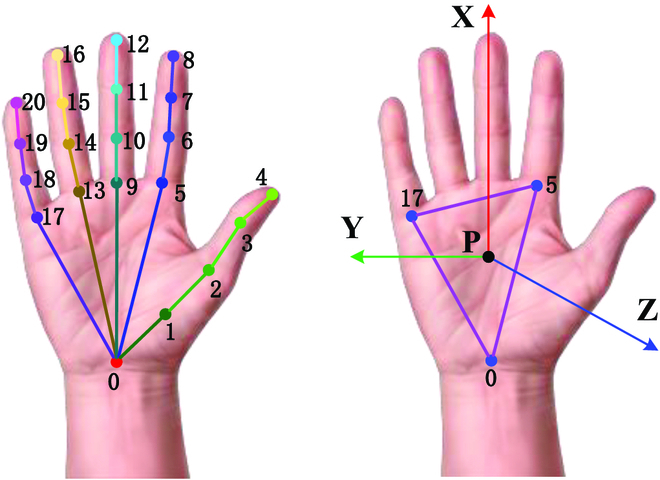
The left image is 3D hand joint points 0 to 20. The right image is the 3D hand orientations calculated by using the planar triangle (0,5,17).

Then, the RGB and depth dual-hand frames are input to the dual-hand motion capture module. The proposed DuHandLocaNet and 3DHandPoseNet are applied to locate and distinguish dual hands and estimate 3D hand pose from the RGB frames. The depth frames are used to obtain the depth information for the 3D hand positions and correct the depth information for the 3D hand pose. In addition, the 3D hand orientations are also required for the bionic bimanual robot teleoperation. We followed the method in [34] to calculate the 3D hand orientations. As shown in Fig. 5, the hand normal vector $\overset{\leftarrow }{Z}$ is calculated as

$$\vec{Z}=\vec{P_{0}P_{5}}\;\times \;\vec{P_{0}P_{17}}$$ (17)

where the vector $\overset{\leftarrow }{Z}$ needs to be normalized to a unit vector, and × is cross product. The hand normal vector $\overset{\leftarrow }{X}$ is calculated as a connection vector from the hand joint point 0 (*P*_0_) to the mean position (*P_M_*) of the hand joint points 5, 9, and 13. The equation is shown as

$$P_{M}=\left\{\begin{array}{ll} \frac{1}{4}\sum ‍P_{i} & i=5,9,13 \end{array}\right.$$ (18)

$$\vec{X}\;=\;\vec{P_{0}P_{M}}$$ (19)

where the vector $\overset{\leftarrow }{X}$ is also normalized to a unit vector. Finally, the hand normal vector is calculated as the cross product of the normalized vector $\overset{\leftarrow }{X}$ and $\overset{\leftarrow }{Z}$.

$$\vec{Y\;}=\;\vec{Z}\times \;\vec{X}$$ (20)

After that, the hand orientation *O^H^* is expressed in a quaternion (*qω*, *qx*, *qy*, *qz*), which is shown as

$$\left\{\begin{array}{c} q\omega =\frac{\sqrt{{\vec{X}}_{x}+{\vec{Y}}_{y}+{\vec{Z}}_{z}}}{2}\\ \mathrm{qx}=\frac{{\vec{Z}}_{y}-{\vec{Y}}_{z}}{4\mathrm{q\omega}}\\ \mathrm{qy}=\frac{{\vec{X}}_{z}-{\vec{Z}}_{x}}{4\mathrm{q\omega}}\\ \mathrm{qz}=\frac{{\vec{Y}}_{x}-{\vec{X}}_{y}}{4\mathrm{q\omega}} \end{array}\right.$$ (21)

### Mapping module

**Fig. 6.**
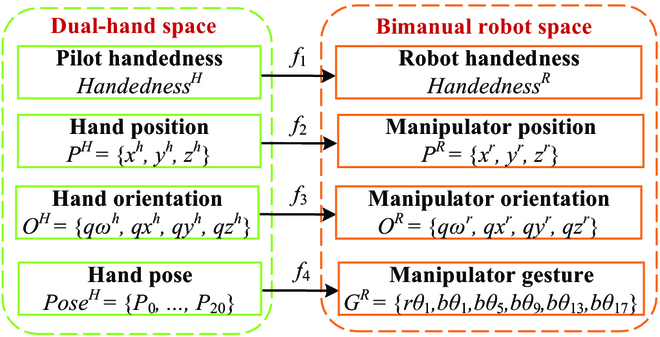
Mapping relationship between the pilot dual hands and bionic bimanual robot.

The mapping relationship between the pilot dual hands and bionic bimanual robot is shown in Fig. 6. Function *f1* maps the pilot handedness to the bionic bimanual robot handedness, which is formulated as

$$\text{Handednes}s^{R}=\left\{\begin{array}{cc} \text{left} & \text{if}\;{\text{Handedness}}^{H}=\text{left}\\ \text{right} & \text{if}\;{\text{Handedness}}^{H}=\text{right} \end{array}\right.$$ (22)

**Fig. 7.**
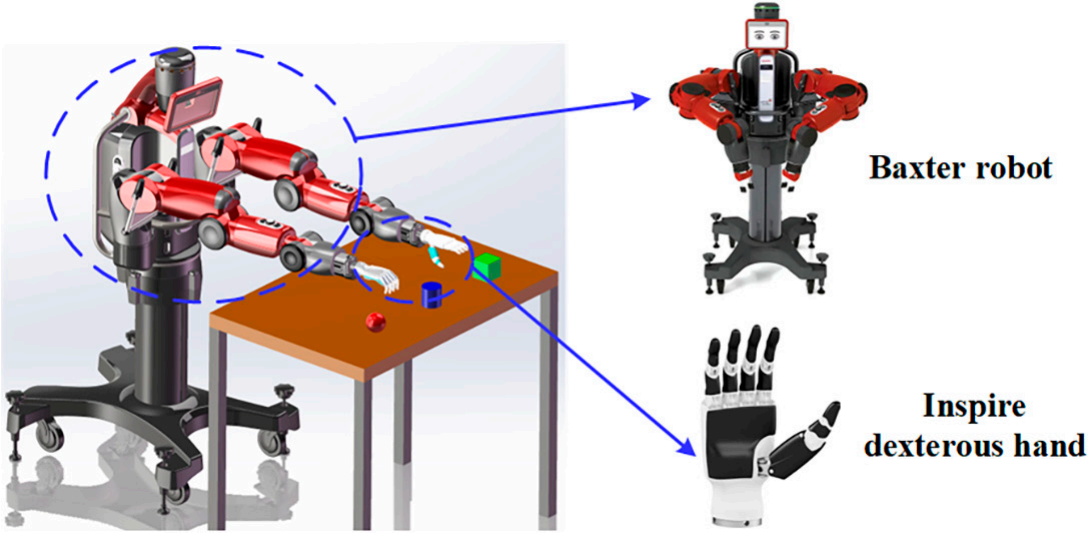
The bionic bimanual robot.

Function *f2* maps the pilot hand position *P^H^* = (*x^h^*, *y^h^*, *z^h^*) to the bionic bimanual robot manipulator position *P^R^* = (*x^r^*, *y^r^*, *z^r^*), which is formulated as

$$\left\{\begin{array}{l} x_{i}^{r}=x_{i-1}^{r}+k_{p}\left(x_{i}^{h}-x_{i-1}^{h}\right)\\ y_{i}^{r}=y_{i-1}^{r}+k_{p}\left(y_{i}^{h}-y_{i-1}^{h}\right)\\ z_{i}^{r}=z_{i-1}^{r}+k_{p}\left(z_{i}^{h}-z_{i-1}^{h}\right) \end{array}\right.$$ (23)

where (*x_i_*, *y_i_*, *z_i_*) is the position in the *i*th frame, and (*x*_*i*−1_, *y*_*i*−1_, *z*_*i* − 1_) is the position in the (*i −* 1)th frame. *k_p_* is the position scaling factor. Function *f3* maps the pilot hand orientation *O^H^* = (*qω^h^*, *qx^h^*, *qy^h^*, *qz^h^*) to the bionic bimanual robot manipulator orientation *O^R^* = (*qω^r^*, *qx^r^*, *qy^r^*, *qz^r^*), which is formulated as

$$\left\{\begin{array}{l} q\omega_{i}^{r}=q\omega_{i-1}^{r}+k_{o}\left(q\omega_{i}^{h}-q\omega_{i-1}^{h}\right)\\ qx_{i}^{r}=qx_{i-1}^{r}+k_{o}\left(qx_{i}^{h}-qx_{i-1}^{h}\right)\\ qy_{i}^{r}=qy_{i-1}^{r}+k_{o}\left(qy_{i}^{h}-qy_{i-1}^{h}\right)\\ qz_{i}^{r}=qz_{i-1}^{r}+k_{o}\left(qz_{i}^{h}-qz_{i-1}^{h}\right) \end{array}\right.$$ (24)

where (*qω_i_*, *qx_i_*, *qy_i_*, *qz_i_*) and (*qω*_*i* −1_, *qx*_*i* −1_, *qy*_*i* −1_, *qz*_*i* −1_) are the orientations in the *i*th and (*i −* 1)th frames, respectively. *k_o_* is the orientation scaling factor. Function *f4* maps the 3D hand pose *Pose^H^* to the manipulator gesture *G^R^* = (*rθ*_1_, *bθ*_1_, *bθ*_5_, *bθ*_9_, *bθ*_13_, *bθ*_17_), which is formulated as

$$r\theta_{1}=k_{g}\cdot \text{arccos}\left(\frac{b_{0,1}^{T}b_{0,5}}{{||b_{0,1}||}_{2}{||b_{0,5}||}_{2}}\right)$$ (25)

$$b\theta_{k}=k_{g}\cdot \text{arccos}\left(\frac{b_{0,k}^{T}b_{k,k+3}}{{||b_{0,k}||}_{2}{||b_{k,k+3}||}_{2}}\right)\;k=1,5,9,13,17$$ (26)

where *rθ*_1_ is the rotational angle of the manipulator thumb, *bθ_k_* are the bending angles of the manipulator 5 fingers. *k_g_* is the angle scaling factor.

### Bionic bimanual robot movement space

The bionic bimanual robot movement space contains a bionic bimanual robot with 2 5-finger dexterous manipulators, which is shown in Fig. 7. Among them, the bionic bimanual robot chooses to use the Baxter robot, which consists of 2 robotic arms with 7 degrees of freedom. The 5-fingered dexterous hand chooses to use the Inspire dexterous hand, which contains 6 degrees of freedom and 21 joints. The movement of the bionic bimanual robot is based on the information output from the mapping module. In addition, the movement status of the bionic bimanual robot can be fed back to pilot space.

## Results and Discussion

In this section, the experiment results and validation of the proposed DuHandLocNet and 3DHandPoseNet are provided in Validation of DuHandLocNet and Validation of 3DHandPoseNet. The experiment details of the dual-hand-based teleoperation for the bionic bimanual robot is shown in Validation of bionic bimanual robot teleoperation.

### Validation of DuHandLocNet

#### Dataset description

For hand detection validation, the public hand datasets Egohands [35] and Oxford Hands [36] were chosen for training and validation. The Egohands contains 4,784 hand RGB images of egocentric interactions with more than 15,000 pixel-level ground-truth hand annotations. Among them, 4,807 images were chosen for training and the remaining 821 images for validation. The Oxford Hands contains 5,628 daily life images with 13,050 hand annotations from public datasets like Buffy Stickman and PASCAL VOC 2007. Among them, 4,807 images and the remaining 821 images were chosen for training and validation.

For dual-hand detection validation, a custom dual-hand detection dataset (DualHands) was used for training and validation. It contains 2000 RGB images collected from 8 samples with 4,000 annotations of “left hand” and “right hand”. These images contain human bodies or upper limbs with different size and hand gestures (American sign language gestures [37]). Both training and validation datasets have 1,000 images.

#### Implementation details

Our experiments were performed on a machine with NVIDIA RTX2080Ti GPU. The Pytorch platform [38] was used for all training and evaluation experiments. For training parameters, batch size was set to 32, initial learning rate was set to 1.25e-4, training epoch was set to 140, and the learning rate dropped by 10 times at epoch 90 and epoch 120, respectively. The accuracy percent with intersection over union (IoU) = [0.50:0.95] was used as the metrics to evaluate the performance of hand detection.

#### Quantitative comparison for hand detection

The comparative experiments for hand detection were conducted on the Egohands [35] and Oxford Hands [36]. The results of the DuHandLocNet were compared with those of some state-of-the-art methods and the compared results are shown in Table 1. The accuracy percent with IoU = [0.50:0.95] is chosen as the metric.

**Table 1.** Compared results for hand detection on the Egohands and Oxford Hands.

Method	Backbone	Egohands	Oxford Hands
Faster R-CNN [17]	Resnet-50	0.730	0.426
SSD300 [18]	VGG-16	0.716	0.392
RetinaNet [44]	ResNet101-FPN	0.768	0.421
YOLO v4 [19]	CSPDarkNet-53	0.732	0.402
CenterNet [29]	DLA-34	0.781	0.438
DuHandLocNet	DLA-34	0.797	0.448

It can be seen from the Table 1 that the proposed DuHandLocNet can achieve 0.797 and 0.448 accuracies on the Egohands and Oxford Hands, respectively. These results are better than that of other state-of-the-art methods. Therefore, it is proved that the DuHandLocNet achieves great hand detection performance. In addition, the hand detection subnetwork in the DuHandLocNet used the CenterNet structure. Compared with the result of the CenterNet (0.781 on the Egohands and 0.438 on the Oxford Hands), the DuHandLocNet improved the accuracies by 0.016 on the Egohands and 0.010 on the Oxford Hands, respectively. It is proved that the proposed parallel network with biological inspiration effectively improves the accuracy of hand detection.

#### Quantitative comparison for dual-hand detection

The comparative experiments for dual-hand detection were conducted on the custom DualHands. The results of the DuHandLocNet were compared with those of some state-of-the-art methods and the compared results are shown in Table 2. The mean average precision with IoU = 0.5 is chosen as the metric.

**Table 2.** Compared results for dual-hand detection on the DualHands.

Method	Left hand	Right hand	Hand
Dual-hand [22]	0.9627	0.9618	0.9734
MS-RFCN [20]	0.9516	0.9458	0.9487
DuHandLocNet	0.9843	0.9786	0.9863

It can be seen from the Table 2 that the proposed DuHandLocNet can achieve 0.9843, 0.9786, and 0.9863 accuracies for left hand, right hand, and hand detection, respectively. These results outperform the results of [20,22]. It is proved that the DuHandLocNet can effectively distinguish between left and right hands by introducing the biological constraint of the structure relationship between the dual hands and body.

**Fig. 8.**
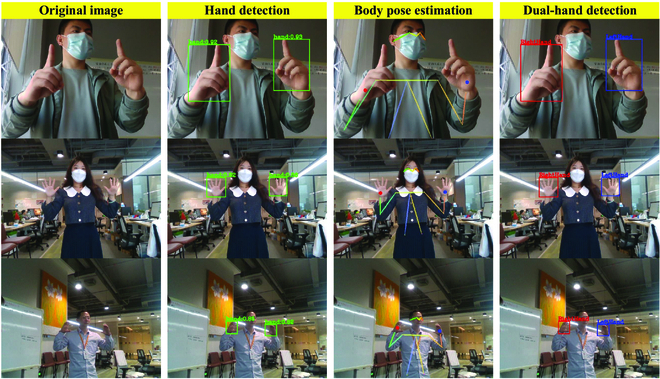
The results of DuHandLocNet. From left to right are original images of pilots with different hand sizes and gestures, hand detection results from the hand detection subnetwork, body pose estimation results from the body pose estimation subnetwork and body FK tree, and dual-hand detection and distinction results from the DuHandLocNet.

To better demonstrate the performance of the DuHandLocNet for dual-hand detection and distinction, some results on DualHands are visualized and shown in Fig. 8. It can be seen from Fig. 8 that the DuHandLocNet performs excellently in detecting and distinguishing the left and right hands with different sizes and gestures.

### Validation of 3DHandPoseNet

#### Dataset description

For 3D hand pose estimation validation, the public datasets Rendered Handpose Dataset (RHD) [39], GANerated Hands Dataset [40], the FreiHAND [41] were chosen for training, which contains 41,258, 330,000, and 130,240 synthetic RGB hand images with 3D hand pose annotations, respectively. The public test datasets RHD [39], Dexter+Object (DO) [42], EgoDexter (ED) [43], and FreiHAND [41] were chosen for evaluation. Among them, the RHD and FreiHAND contain 2,728 and 3,960 evaluation samples. The DO and ED contain 6 sequences from a third view and 4 sequences from an egocentric view.

#### Implementation details

For training parameters, batch size was set to 8, initial learning rate was set to 10-3, training epoch was set to 100, and the learning rate dropped by 10 times at epoch 30 and epoch 60, respectively. The factors of the bone-constraint loss function *λ_hm_*, *λ_pr_*, *λ_len_*, and *λ_dir_* were set to 0.1, 1, 0.001, and 0.1, respectively. The percentage of correct 3D keypoints (PCK) and the area under the PCK curve (AUC) with thresholds ranging from 20 to 50 mm were used as the metrics to evaluate the performance of 3D hand pose estimation.

#### Quantitative comparison for 3D hand pose estimation

The comparison experiments for 3D hand pose estimation were evaluated on the RHD, DO, and ED datasets. The results of the proposed 3DHandPoseNet were compared with some of the state-of-the-art methods, which are shown in Table 3. In Table 3, the AUC with thresholds ranging from 20 to 50 mm is chosen as the metric. “∗” denotes that the model was trained on this dataset, and “-” denotes that no results were reported on this dataset. In addition, it is necessary to notice that not all the compared methods were trained on the same datasets.

**Table 3.** Compared experiment results for 3D hand pose estimation.

Method	DO	ED	RHD
Boukhayma et al. [45]	0.763	0.674	-
Xiang et al. [46]	0.912	-	-
Baek et al. [47]	0.650	-	0.926*
Zhang et al. [48]	0.825	-	0.901*
Ge et al. [24]	-	-	0.920*
Zhou et al. [49]	0.948	0.811	0.856*
Kourbane et al. [50]	-	-	0.915*
3DHandPoseNet	0.952	0.927	0.934*

It can be seen from the Table 3 that the proposed 3DHandPoseNet can achieve 0.952, 0.927, and 0.934 accuracies on the DO, ED, and RHD datasets, respectively. These results outperform the results of the state-of-the-art methods. Therefore, it is proved that the 3DHandPoseNet can effectively estimate the 3D hand pose.

#### Ablation study for 3DHandPoseNet

**Fig. 9.**
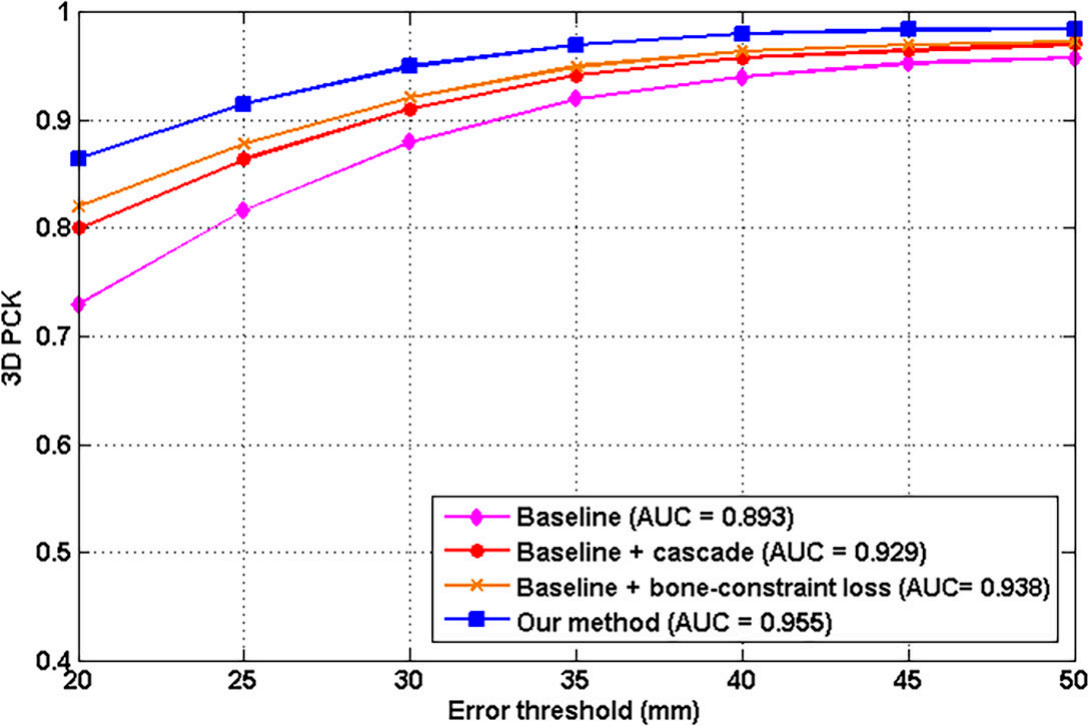
Ablation experiment results on the FreiHAND dataset.

The ablation experiments for 3D hand pose estimation were evaluated on the FreiHAND dataset. Four models were evaluated for comparison. (a) As shown in Fig. 4, the backbone of the first model used 2 hourglass modules. Then, a residual block with max pooling was followed to refine the features and 2 full-connected layers with dropout were followed to regress 3D hand pose. This model was set as the baseline. (b) Based on the baseline, the proposed cascade structure was added following Fig. 4 in the second model. (c) Based on the baseline, the proposed bone-constraint loss functions were added following Fig. 4 in the third model. (d) Based on the baseline, both cascade structure and bone-constraint loss functions were added in the fourth model, which is the 3DHandPoseNet. The 3D PCK is used as the metric. The results are shown in Fig. 9.

It can be seen from the Fig. 9 that the 3D PCK of the second model (0.929), the third model (0.938), and the 3DHandPoseNet (0.955) were improved by 0.036, 0.045, and 0.062 compared with that of the baseline (0.893). These results prove that the proposed cascade structure and bone constraint loss functions can effectively improve the 3D hand pose estimation.

**Fig. 10.**
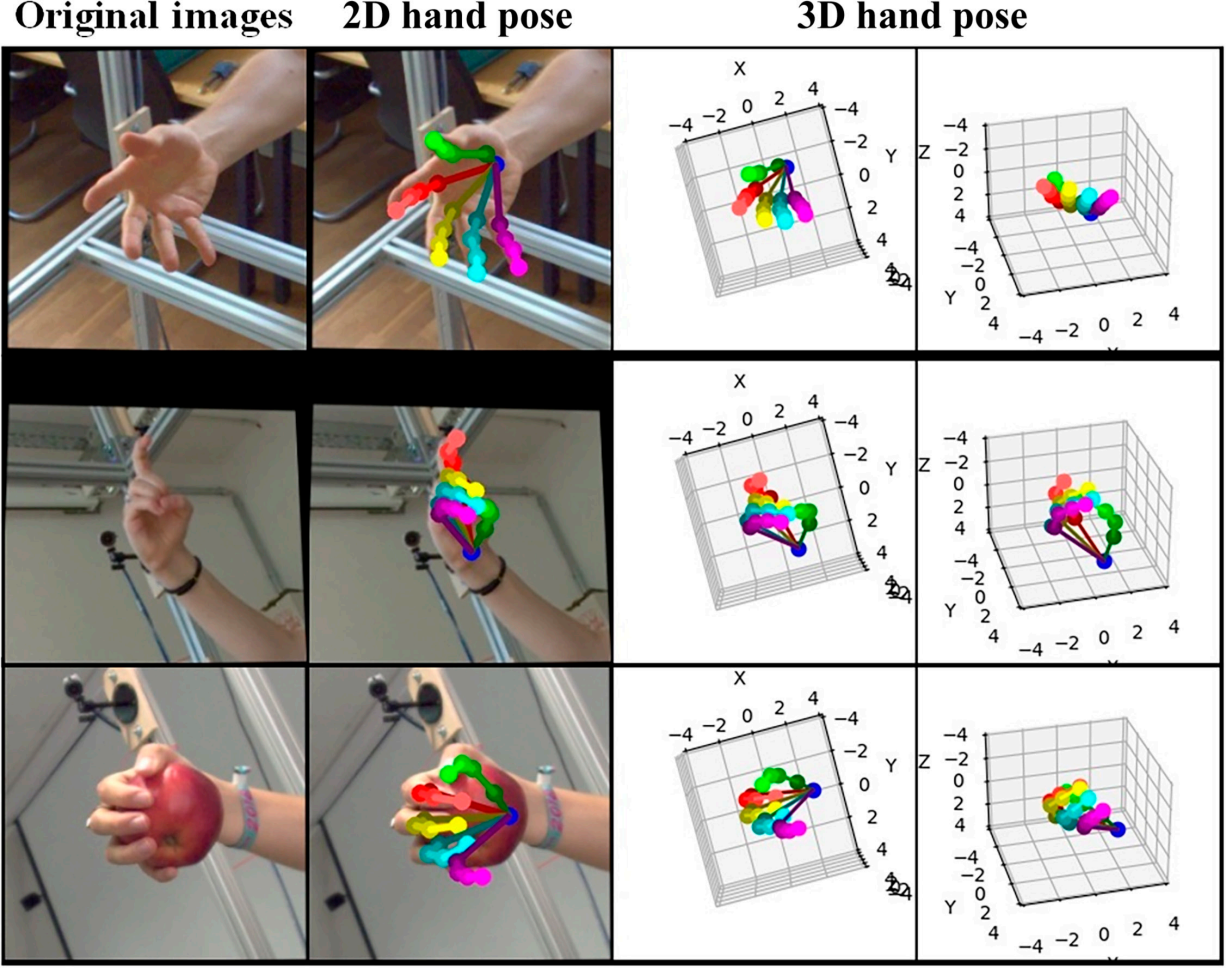
The visualization of 3DHandPoseNet. From left to right are original images, output 2D hand poses, and output 3D hand poses from the camera viewpoint and output 3D hand poses from another viewpoint.

To better demonstrate the effectiveness of the proposed 3DHandPoseNet, some experiment results are visualized in Fig. 10.

### Validation of bionic bimanual robot teleoperation

**Fig. 11.**
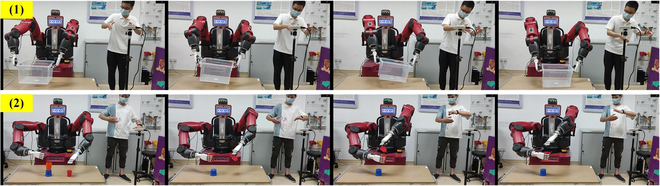
Bionic bimanual robot teleoperation experiments and results. (1) Carrying a big box with dual hands. (2) Inserting a cup into another cup with dual hands.

To verify the performance of the proposed bionic bimanual robot teleoperation, 2 types of physical tasks including box carry and cup insertion were conducted to test the bimanual teleoperation. For the box carry task, the bimanual robot carried the box and moved on the table from one place to another using its dual arms. This task makes a great challenge to the coordination between 2 arms. For the cup insertion task, a smaller cup in the left hand is inserted into a larger cup in the right hand, which makes a challenge to operation accuracy requirements. Before this experiment, 2 pilots went through a warm-up training phase. Next, each pilot conducted 3 consecutive test trials for each task as shown in Fig. 11. Both tasks got 100% success accuracy because the object dropped in one of the experiments. The time spent on the box carry task is within 100 s, while the cup insertion task is within 90 s. The teleoperation tracking error is about 9.3 mm, and the delay time is about 0.68 s. These results show that the proposed dual-hand-based bionic bimanual robot teleoperation method has good performance in the dexterity, accuracy, and coordination of operation.

## Conclusion

In this paper, to deal with the bionic bimanual robot teleoperation, a visual dual-hand motion capture based on biological inspiration and a dual-hand-based bionic bimanual robot teleoperation method were proposed. The contributions and innovations are summarized as follows. (a) A DuHandLocNet was proposed to deal with dual-hand detection and localization, which introduced the biological constraint of the structure relationship between the dual hands and body by using a parallel network. (b) A 3DHandPoseNet was proposed to deal with 3D hand pose estimation from single RGB images, which was based on the hand biological inspiration by using a cascaded structure and a bone-constraint loss. (c) A visual dual-hand-based teleoperation system for bionic bimanual robots was designed by using the proposed DuHandLocNet and 3DHandPoseNet. Experiments on public hand datasets demonstrated the effectiveness and superiority of proposed DuHandLocNet and 3DHandPoseNet. Experiments on a bionic bimanual robot platform demonstrated that the proposed teleoperation method can realize free and convenient teleoperation.

In the future, (a) more robust and accurate dual-hand motion capture methods should be researched. The video-based hand detection and hand pose estimation method will be used to improve the accuracy and robustness of the hand motion capture. (b) MixedReality and shared control method will be applied to the bionic bimanual robot teleoperation to increase teleoperation immersion and efficiency.

## Data Availability

The data that support the findings of this study are available from the corresponding author upon reasonable request.
